# Heat-Induced Phytochemical Changes in *Curcuma longa*: Effects on Antioxidant Properties and Oxidative Behavior in Illuminated Oil-in-Water Emulsions

**DOI:** 10.3390/antiox15081030

**Published:** 2026-08-19

**Authors:** Choong-In Yun, Juhee Cho, Ji-Won Jeong, Young-Jun Kim, JaeHwan Lee

**Affiliations:** 1 Department of Food Science and Biotechnology, Sungkyunkwan University, Suwon 16419, Republic of Korea; innny@skku.edu (C.-I.Y.); kellly33@skku.edu (J.-W.J.); 2 Research Institute of Food and Biotechnology, Seoul National University of Science and Technology, Seoul 01811, Republic of Korea; 3 Department of Food Science and Biotechnology, Seoul National University of Science and Technology, Seoul 01811, Republic of Korea; whwngml19992@seoultech.ac.kr (J.C.)

**Keywords:** *Curcuma longa*, thermal processing, curcuminoids, sesquiterpenoids, oxidative stability

## Abstract

*Curcuma longa* is a medicinal plant valued for its diverse phytochemicals and antioxidant properties. However, the effects of thermal processing on its phytochemical composition and antioxidant function remain insufficiently understood. This study investigated heat-induced changes in curcuminoids, sesquiterpenoids, and their degradation products in *C. longa* extracts heated at 180 °C for 0–90 min under dry, aqueous, olive oil, and corn oil conditions using UHPLC–MS/MS and GC–MS. Curcuminoids progressively decreased during heating, whereas sesquiterpenoids exhibited greater thermal stability, particularly in oil matrices. Antioxidant properties were also better preserved in oil-treated extracts than in those heated under dry or aqueous conditions. However, in an oil-in-water emulsion system exposed to continuous LED illumination (14,600 lux), extracts obtained after dry heating accelerated lipid oxidation, as evidenced by enhanced oxygen depletion, hydroperoxide formation, and conjugated diene accumulation. This pro-oxidative effect was markedly suppressed by EDTA, suggesting the involvement of metal-mediated oxidation pathways. These findings demonstrate that thermal processing differentially alters the phytochemical profile and antioxidant properties of *C. longa* depending on the heating matrix, while highlighting that preserved antioxidant capacity does not necessarily translate into improved oxidative stability in illuminated emulsion systems.

## 1. Introduction

*Curcuma longa* L. (turmeric) is a medicinal plant widely used in foods and traditional medicine because of its diverse phytochemicals and associated biological activities. Its major bioactive constituents comprise two principal phytochemical groups: curcuminoids, including curcumin, demethoxycurcumin, and bisdemethoxycurcumin, and volatile essential oils dominated by sesquiterpenoids such as *ar-*turmerone, α-turmerone, and bisacurone [1,2,3]. Curcuminoids are recognized as the primary contributors to the antioxidant activity of turmeric, whereas sesquiterpenoids possess complementary antioxidant, anti-inflammatory, and other biological properties [4,5,6]. Consequently, the functional properties of turmeric depend on the combined contributions of multiple phytochemical classes rather than on curcuminoids alone.

Thermal processing is an inevitable step during the manufacture, cooking, and utilization of turmeric-containing products, and it can substantially alter phytochemical composition. Curcuminoids readily undergo thermal and oxidative degradation, producing lower-molecul*ar-*weight phenolic compounds such as vanillin, vanillic acid, ferulic acid, and dehydrozingerone, some of which retain antioxidant activity [2,3,7]. In contrast, essential oil constituents generally exhibit greater thermal stability than curcuminoids, although their stability varies depending on processing conditions and surrounding environments [5,8,9]. These compositional changes may alter the balance between native phytochemicals and newly generated degradation products, thereby influencing the overall antioxidant properties of turmeric extracts.

The extent of these transformations is also affected by the surrounding matrix. Previous studies have demonstrated that aqueous and lipid environments differently influence the degradation, partitioning, and stability of curcumin and related compounds [10,11]. However, most investigations have focused primarily on curcuminoids, whereas the simultaneous thermal responses of sesquiterpenoids and degradation products have received considerably less attention. Furthermore, it remains unclear whether matrix-dependent phytochemical changes are reflected in the oxidative behavior of complex food systems, such as oil-in-water (O/W) emulsions, where lipid oxidation is strongly governed by interfacial reactions [12,13].

Korean *C. longa* (Ulgeum) has been reported to exhibit a distinct phytochemical composition, with relatively higher levels of sesquiterpenoids and lower concentrations of curcuminoids than turmeric samples from several other geographical origins [9,14]. However, such compositional variation cannot be attributed to climatic conditions alone, as phytochemical profiles may also be influenced by genetic background, geographical origin, cultivation practices, harvest conditions, and postharvest processing. Accordingly, the compositional characteristics reported for Korean *C. longa* should be interpreted as a region-associated phytochemical profile rather than a climate-defined chemotype. Such compositional characteristics suggest that Korean turmeric may exhibit thermal responses different from those reported for conventional curcuminoid-dominant turmeric, particularly with respect to the balance among curcuminoids, sesquiterpenoids, and their thermally generated degradation products.

Therefore, this study comprehensively investigated matrix-dependent heat-induced phytochemical changes in Korean *C. longa* by simultaneously quantifying curcuminoids, sesquiterpenoids, and their degradation products under dry, aqueous, and oil-based heating conditions. Changes in antioxidant properties were evaluated using complementary chemical assays, and their functional implications were further examined in an illuminated oil-in-water emulsion model to determine how thermal processing influences the oxidative behavior of turmeric extracts under application-relevant conditions.

## 2. Materials and Methods

### 2.1. Reagents and Standards

#### 2.1.1. Analytical Standards and Chromatographic Solvents

Authentic standards of curcumin, demethoxycurcumin, bisdemethoxycurcumin, and vanillin-^13^C_6_ were purchased from Toronto Research Chemicals (Toronto, ON, Canada). Bisacurone, *ar-*turmerone, vanillin, and vanillylidenacetone (dehydrozingerone) were obtained from Sigma-Aldrich (St Louis, MO, USA). For GC–MS, ethyl octanoate (≥99%; Sigma-Aldrich) was used as the internal standard (IS), and an n-alkane standard mixture (C8–C20, 40 mg/L each; Sigma-Aldrich) was used for retention index (RI) calibration. HPLC-grade solvents (methanol, n-hexane, acetonitrile, absolute ethanol, and water) were from J.T. Baker (Phillipsburg, NJ, USA); LC–MS grade formic acid was from Sigma-Aldrich.

#### 2.1.2. Antioxidant and Oxidative Stability Reagents

Antioxidant reagents (DPPH, ABTS, 2,2′-azobis(2-amidinopropane) dihydrochloride [AAPH], fluorescein sodium salt, Trolox, gallic acid, catechin, Folin–Ciocalteu reagent, aluminum chloride [AlCl_3_], acetic acid, 2,4,6-tri(2-pyridyl)-1,3,5-triazine [TPTZ], ferric chloride [FeCl_3_], and ferrous sulfate [FeSO_4_]) were from Sigma-Aldrich. Sodium nitrite, sodium carbonate (Na_2_CO_3_), hydrochloric acid (HCl), and sodium acetate were from Junsei Chemical Co. (Tokyo, Japan). For oil stripping and emulsion preparation, silicic acid (acid-washed, 100–200 mesh), activated charcoal (100 mesh, decolorizing grade), polyoxyethylene sorbitan monolaurate (Tween 20), and disodium ethylenediaminetetraacetate dihydrate (Na_2_EDTA·2H_2_O) were supplied by Sigma-Aldrich. Oxidative stability reagents (1-butanol, isooctane, 2-propanol, barium chloride [BaCl_2_], and ammonium thiocyanate [NH_4_SCN]) were also from Sigma-Aldrich, while 0.1 M HCl was purchased from Wako Pure Chemical Industries (Osaka, Japan).

### 2.2. Preparation of Stripped Edible Oils

Refined corn and olive oils were stripped of endogenous minor components, including tocopherols and pigments, using a modified adsorption-based chromatographic method [15]. Briefly, oils (40 g) were dissolved in n-hexane (1:1, *w*/*v*) and passed through a glass column packed with acid-washed silicic acid and activated charcoal. The neutral lipid fraction (~400 mL) was eluted with n-hexane, then evaporated under reduced pressure using a rotary evaporator, and stored at −80 °C in sealed containers. All steps were performed under chilled, light-protected conditions to prevent unintended oxidation. The effectiveness of the stripping procedure was verified by comparing the DPPH radical-scavenging activity of the oils before and after stripping, according to the method of La et al. [16]. The markedly reduced DPPH radical-scavenging activity after stripping confirmed the effective removal of endogenous free radical-scavenging compounds, including tocopherols.

### 2.3. Plant Material and Thermal Processing

#### 2.3.1. Species Authentication

Dried *C. longa* (Ulgeum) harvested in South Korea was authenticated by genomic DNA extraction (CTAB method) and PCR amplification of the internal transcribed spacer 2 (ITS2) region and chloroplast trnK/matK locus using primers described by [17]. Sequence similarity was evaluated using BLASTn software (Version 2.13.0) analysis against the NCBI GenBank database. The resulting sequences showed 99.9–100% identity with previously deposited, authenticated *C. longa* reference sequences in GenBank (e.g., MW838984.1 and AB047738.1). These results, consistent with our previous report [18], confirmed the taxonomic identity of the samples.

#### 2.3.2. Thermal Treatment

Powdered samples (0.5 g) were subjected to four heating conditions: dry, aqueous, olive oil, and corn oil. No additional matrix was used for the dry condition. For the aqueous condition, 5.0 g of distilled water was added to the powder (1:10, *w*/*w*), whereas 0.5 g of olive oil or corn oil was added for the respective oil-based conditions (1:1, *w*/*w*). The samples were distributed as a thin layer at the bottom of 50 mL amber volumetric flasks. The flasks were closed with their caps but were not hermetically sealed, allowing vapor release during heating. The laboratory oven was preheated to 180 °C before sample introduction. Flasks assigned to the 10, 30, 60, and 90 min treatments were placed in the preheated oven simultaneously and individually removed at their designated heating times; the 0 min samples served as unheated controls. Parameters were selected based on known curcuminoid degradation kinetics [2]. Substantial water loss occurred during prolonged heating under the aqueous condition, with visible partial charring observed after 90 min. Samples were cooled to ambient temperature prior to extraction.

### 2.4. Extraction Procedures

#### 2.4.1. Methanolic Extraction for UHPLC–MS/MS, Antioxidant Activity, and Oxidative Stability Assays

Heat-treated powders (0.5 g) were extracted with 50 mL HPLC-grade methanol by ultrasonic irradiation (50 kHz, 500 W), as described by [9], with minor modifications. Extracts were centrifuged, filtered (0.22 μm regenerated cellulose; Sartorius, Göttingen, Germany), and evaporated to dryness under nitrogen at 40 °C. For the antioxidant assays, a 2 mL aliquot of the methanolic extract was evaporated completely under nitrogen at 40 °C and reconstituted with 2 mL of distilled water. The reconstituted extracts were subsequently diluted 80–100-fold for the DPPH, FRAP, and ORAC assays and 5–10-fold for the ABTS assay using the same solvent system employed for the corresponding calibration standards. For emulsion experiments, methanolic extracts (50 mL) were concentrated under nitrogen at 40 °C to a final volume of 10 mL and used for incorporation into the O/W emulsion system. All analytical results were calculated relative to the initial dry mass of *C. longa* and are expressed per kilogram of dried *C. longa*, irrespective of the amount of water or oil used as the heating matrix.

#### 2.4.2. Hexane Extraction for GC–MS Analysis

For volatile profiling, powders were extracted with 50 mL n-hexane by ultrasonic irradiation (same conditions as in Section 2.4.1). Extracts were filtered (0.22 μm membrane) prior to GC–MS analysis. Ethyl octanoate was used as the IS to improve repeatability: a 100 mg/L stock in n-hexane was prepared, and 50 μL of IS solution was added to 950 μL of each extract. Mixtures were vortexed to ensure homogeneity before injection.

### 2.5. Preparation of Standards and Quantitative Analysis by UHPLC–MS/MS

#### 2.5.1. Quantification of Curcuminoids and Sesquiterpenoids

Stock solutions of curcumin, demethoxycurcumin (DMC), bisdemethoxycurcumin (BDMC), *ar-*turmerone, and bisacurone were prepared in methanol. Working calibration solutions were prepared by serial dilution in methanol, with calibration ranges of 0.002–0.5 μg/mL for curcumin, DMC, BDMC, and *ar-*turmerone and 0.004–1.0 μg/mL for bisacurone. Quantification was performed using external calibration without an internal standard. Methanolic *C. longa* extracts were diluted with methanol as appropriate to maintain the analyte concentrations within the respective calibration ranges prior to injection.

Quantitative analysis was performed using a Vanquish Flex UHPLC (Thermo Fisher Scientific, San Jose, CA, USA) coupled to a TSQ Altis™ Plus triple quadrupole mass spectrometer (Thermo Fisher Scientific, Waltham, MA, USA), using the previously validated UHPLC–MS/MS method reported by Yun et al. (2025) [19]. The method was previously validated for linearity, accuracy, precision, matrix effects, LOD, and LOQ in *C. longa* extracts. Curcuminoids and sesquiterpenoids were separated on a ZORBAX Eclipse Plus C18 column (2.1 × 50 mm, 1.8 μm; Agilent Technologies, Berks, UK) under optimized reversed-phase conditions. Detection was performed in multiple reaction monitoring (MRM) mode with heated electrospray ionization in positive-ion mode. Detailed chromatographic and MS/MS parameters are provided in Tables S1 and S3.

#### 2.5.2. Quantification of Thermal Degradation Products

The quantitative method for vanillin and dehydrozingerone was also previously validated in *C. longa* extracts for linearity, accuracy, precision, matrix effects, LOD, and LOQ [19]. Vanillin and dehydrozingerone were quantified using a separate UHPLC–MS/MS method. Stock solutions of vanillin and dehydrozingerone were prepared in methanol at 500 μg/mL. Working calibration solutions were prepared by serial dilution, covering 0.1–10 μg/mL for vanillin and 0.01–2 μg/mL for dehydrozingerone. Vanillin-^13^C_6_ was used as the internal standard specifically for the analysis of these thermal degradation products. Methanolic *C. longa* extracts were mixed 1:1 (*v*/*v*) with 0.2% formic acid in water and filtered prior to injection. Further dilution was performed as necessary to ensure that analyte concentrations were within the respective calibration ranges.

Analysis was performed using the same UHPLC–MS/MS system described in Section 2.5.1. Vanillin and dehydrozingerone were separated on an Imtakt Unison UK C18 column (2.0 × 100 mm, 3 μm; Imtakt, Kyoto, Japan) under chromatographic conditions modified from [20]. Detection was performed in MRM mode with heated electrospray ionization, using negative-ion mode for vanillin and positive-ion mode for dehydrozingerone and vanillin-^13^C_6_. Detailed chromatographic and MS/MS parameters are provided in Tables S2 and S3.

### 2.6. GC–MS Profiling of Essential Oil Volatiles

Volatile constituents in hexane extracts were analyzed by GC–MS under conditions adapted from established turmeric profiling methods [9,14]. Analyses were performed on an Agilent 7890B gas chromatograph coupled to a 5977B mass selective detector (Agilent Technologies, Inc., Santa Clara, CA, USA) with a DB-5MS UI capillary column (30 m × 0.25 mm × 0.25 μm; Agilent Technologies, Santa Clara, CA, USA). Helium was used as the carrier gas (1.0 mL/min). Samples (1 μL) were injected in split mode (10:1) at an injector temperature of 280 °C. The oven was held at 100 °C for 3 min, ramped at 5 °C/min to 150 °C, then increased to 156 °C at 1 °C/min and held for 4 min, followed by an increase to 166 °C at 2.5 °C/min. The temperature was subsequently increased to 220 °C at 15 °C/min and held for 5 min. A post-run temperature of 300 °C was applied for 5 min. Ion source and transfer line temperatures were set at 230 and 280 °C, respectively. Electron ionization (EI) was at 70 eV, with mass spectra acquired in full scan mode (*m*/*z* 50–550). Olive oil-only and corn oil-only controls were subjected to the same extraction procedure and analyzed by GC–MS; none of the volatile compounds included in the semi-quantitative analysis were detected in either oil control.

Volatile identification was performed using the NIST MS Search Program (v2.3) with the NIST17 Mass Spectral Library (National Institute of Standards and Technology, Gaithersburg, MD, USA). Compounds were identified by comparison of mass spectra with library entries and by evaluation of RIs. The NIST library match factors of the identified compounds ranged from 800 to 967. RIs were calculated relative to a homologous series of n-alkanes (C8–C20) analyzed under identical conditions. Experimental RI values were compared with published values [21,22,23,24], with absolute differences of 0–8 RI units for the identified compounds.

#### Semi-Quantitative Determination Using *ar*-Turmerone Equivalents

Semi-quantitative analysis of volatile constituents was performed using *ar-*turmerone as a reference standard. Calibration solutions of *ar-*turmerone were prepared in n-hexane. Ethyl octanoate was used as the IS to correct for injection variability; a fixed amount was added to each sample prior to GC–MS analysis. Calibration curves were constructed by plotting the peak area ratio of *ar-*turmerone to IS versus concentration. The relative response of each identified compound was then expressed as *ar-*turmerone equivalents, and the resulting concentrations were considered estimates because compound-specific response factors were not determined. Differences in GC–MS response among structurally different volatile compounds may therefore affect the estimated concentrations. Results are reported as estimated milligrams of *ar-*turmerone equivalents per kilogram of sample.

### 2.7. Determination of Total Phenolics, Flavonoids, and Radical-Scavenging Activities

Total phenolic content (TPC), total flavonoid content (TFC), and radical-scavenging capacities (DPPH, ABTS, oxygen radical absorbance capacity [ORAC], ferric reducing antioxidant power [FRAP]) were determined from extracts by spectrophotometric or fluorometric assays. All measurements were performed in triplicate. In addition, extract-specific blanks were used to correct for background absorbance due to the intrinsic color and turbidity of the extracts. Calibration curves were constructed using appropriate standards. Results were expressed on a dry-weight basis: TPC as millimoles gallic acid equivalents per kilogram (mmol GAE/kg); TFC as millimoles catechin equivalents per kilogram (mmol CE/kg); DPPH, ABTS, and ORAC as millimoles Trolox equivalents per kilogram (mmol TE/kg); and FRAP as millimoles FeSO_4_ equivalents per kilogram (mmol FeSO_4_ Eq/kg).

#### 2.7.1. Total Phenolic Content (TPC)

TPC was measured using the Folin–Ciocalteu assay [25] with minor modifications. Extract (0.2 mL) was mixed with Folin–Ciocalteu reagent (0.2 mL) and distilled water (2.6 mL). After equilibration, sodium carbonate (7%, 2 mL) was added. The mixture was incubated at room temperature in the dark for 90 min, and absorbance was read at 750 nm (V-730, Jasco Inc., Tokyo, Japan). Results were calculated from a gallic acid calibration curve.

#### 2.7.2. Total Flavonoid Content (TFC)

TFC was determined by the aluminum chloride colorimetric method [25] with minor modifications. Extract (1 mL) was sequentially reacted with sodium nitrite (5%, 0.3 mL), aluminum chloride (10%, 0.3 mL), and sodium hydroxide (1 M, 2 mL), then diluted to 10 mL with distilled water. Absorbance was read at 510 nm. Quantification used a catechin calibration curve.

#### 2.7.3. DPPH Radical-Scavenging Activity

DPPH activity was assessed as described by [25] with minor modifications. Extract (50 μL) was added to 2.95 mL DPPH solution (100 μM in 80% methanol), incubated for 30 min in the dark, and absorbance was read at 517 nm. Results were calculated from a Trolox calibration curve.

#### 2.7.4. ABTS Radical-Scavenging Activity

ABTS activity was measured following [25] with minor modifications. Extract (20 μL) was added to 980 μL ABTS solution (initial A734 = 0.63–0.67), incubated at 37 °C for 10 min, and absorbance was measured at 734 nm. Results were calculated from a Trolox calibration curve.

#### 2.7.5. Oxygen Radical Absorbance Capacity (ORAC)

ORAC was determined using a fluorescence microplate assay [26] with minor modifications. Extract or Trolox (25 μL) was mixed with fluorescein (150 μL), pre-incubated (37 °C, 30 min, dark), then AAPH (25 μL) was added. Fluorescence (Ex 485 nm/Em 528 nm) was measured every minute for 60 min. Antioxidant capacity was calculated from the area under the curve (AUC) and a Trolox calibration curve.

#### 2.7.6. Ferric Reducing Antioxidant Power (FRAP)

FRAP was measured according to [4], with minor modifications. Extract (0.2 mL) was mixed with FRAP reagent (1.5 mL), incubated at room temperature in the dark for 4 min, and absorbance was read at 593 nm. Results were calculated from a ferrous sulfate calibration curve.

### 2.8. Oxidative Stability Testing in Emulsion Systems

The oxidative stability of *C. longa* extracts was assessed using a 2.5% (*w*/*w*) O/W emulsion model. Only extracts from dry thermal treatment were evaluated. Emulsions were used to accelerate lipid oxidation and compare the protective effects of extracts subjected to different heating times. Oxidative progression was monitored by measuring headspace oxygen depletion, lipid hydroperoxide formation, and conjugated diene (CD) development.

#### 2.8.1. Oil-in-Water (O/W) Emulsion Model

Emulsions (nominally 2.5%, *w*/*w* soybean oil) were prepared according to [27], with minor modifications. Distilled water (624 mL) was combined with 5 mL of each test solution: blank (methanol), standard (Trolox, 6.26 mg/mL; 25 mmol TE/kg), or methanolic *C. longa* extracts from samples heated at 180 °C for 0, 10, 30, 60, or 90 min. The amount of extract added was standardized based on the 0-min (unheated) extract, corresponding to an ORAC activity of 25 mmol TE/kg, and the same turmeric-equivalent amount was applied to all heat-treated extracts. Soybean oil (16 g) and Tween 20 (1.6 g) were added as the lipid phase and emulsifier. The mixture was homogenized (HB501, Tefal, Haute-Savoie, France) for 3 min, then nano-dispersed (ISA-LM100, Ilshinautoclave Co., Ltd., Daejeon, Republic of Korea) at 27 MPa for three cycles. Aliquots (2 mL) of each emulsion were sealed in 10 mL glass vials and stored at 25 °C for 8 days under continuous white LED illumination (14,600 lux) to induce photo-oxidation. Oxidative changes were monitored periodically.

#### 2.8.2. Headspace Oxygen Analysis

Oxygen depletion was monitored as described by [27,28], with minor modifications. Separate vials were prepared for each sampling time point, and each vial was used only once for headspace oxygen analysis. At each sampling, 40 μL of headspace gas was withdrawn using a gas-tight syringe and injected into a GC–TCD (Hewlett-Packard 7890, Agilent Technologies, Inc., Santa Clara, CA, USA) fitted with a 60/80-mesh packed column (3.0 m × 2 mm i.d.; Restek, Bellefonte, PA, USA) and thermal conductivity detector. Helium (200 mL/min) was the carrier gas. Oven temperature was 60 °C; injector and detector were set to 180 °C.

#### 2.8.3. Lipid Hydroperoxide Analysis

Lipid hydroperoxides were quantified using a modified ferric thiocyanate method [27]. Emulsion (0.3 mL) was mixed with isooctane/2-propanol (3:1, *v*/*v*; 1.5 mL), vortexed (1 min), and centrifuged (3 min). The upper organic phase (0.2 mL) was combined with methanol/1-butanol (2:1, *v*/*v*; 2.8 mL); 1 mL was reacted with 10 μL of freshly prepared 0.072 M Fe^2+^ solution. Vortexed samples were incubated in the dark at room temperature for 20 min, and absorbance was measured at 510 nm. Samples were further diluted as needed (up to 30-fold) to maintain absorbance within the linear range, and the corresponding dilution factors were applied to the final calculations. Concentrations were calculated from a cumene hydroperoxide calibration curve (0.3125–5.000 mM, R^2^ = 0.9974) and expressed as mmol hydroperoxide equivalents/kg oil.

#### 2.8.4. Conjugated Diene (CD) Analysis

CDs were measured spectrophotometrically to monitor primary lipid oxidation. Emulsion (120 μL) was mixed with methanol/1-butanol (2:1, *v*/*v*; 2.7 mL), vortexed, and absorbance was read at 233 nm in a quartz cuvette. Samples were further diluted with the same solvent mixture as needed (up to 30-fold) to maintain absorbance within the linear range, and the corresponding dilution factors were applied to the final calculations. CD content was calculated using the equation CD (g/100 g oil) = 1.0769 × A_233_/C, where A_233_ is the absorbance at 233 nm and C is the oil concentration (g/L) in the measured solution. The resulting values were converted and expressed as grams of conjugated dienes per kilogram of oil (g/kg oil).

#### 2.8.5. Effect of EDTA on Oxidative Stability

To evaluate the potential involvement of metal-mediated oxidation, additional O/W emulsions were prepared with EDTA. EDTA was dissolved in 624 mL of distilled water to obtain a concentration of 0.1 mM before emulsion preparation. The EDTA-containing aqueous phase was then combined with 5 mL of the methanolic *C. longa* extract, 16 g of soybean oil, and 1.6 g of Tween 20, following the same emulsification procedure described in Section 2.8.1. Extracts obtained after 0 and 30 min of dry thermal treatment were evaluated with and without EDTA. The resulting emulsions were stored and analyzed for headspace oxygen, lipid hydroperoxides, and conjugated dienes under the same conditions described above.

### 2.9. Statistical Analysis

All experiments were independently performed in triplicate (*n* = 3), including thermal treatment and extraction as well as preparation of the O/W emulsions, and the results are reported as mean ± standard deviation. For the thermal-treatment experiments, two-way analysis of variance (ANOVA) was used to evaluate the effects of heating matrix, heating time, and their interaction. For the O/W emulsion experiments, two-way ANOVA was used to evaluate the effects of treatment, storage time, and their interaction. When significant effects were observed, Tukey’s post hoc test was used for multiple comparisons as appropriate. Statistical significance was set at *p* < 0.05. Pearson’s correlation coefficients were calculated to assess associations between quantified phytochemicals, TPC, TFC, and antioxidant indices (DPPH, ABTS, ORAC, FRAP). For the correlation analysis, mean values from 20 experimental conditions (four heating matrices × five heating times; *n* = 20) were used, and *p*-values were adjusted for multiple comparisons using the Benjamini–Hochberg false discovery rate (FDR) procedure.

## 3. Results and Discussion

### 3.1. Matrix-Dependent Alterations in Major Bioactive Compounds and Thermal Degradation Products of C. longa Extracts Determined by UHPLC–MS/MS

**Figure 1:**
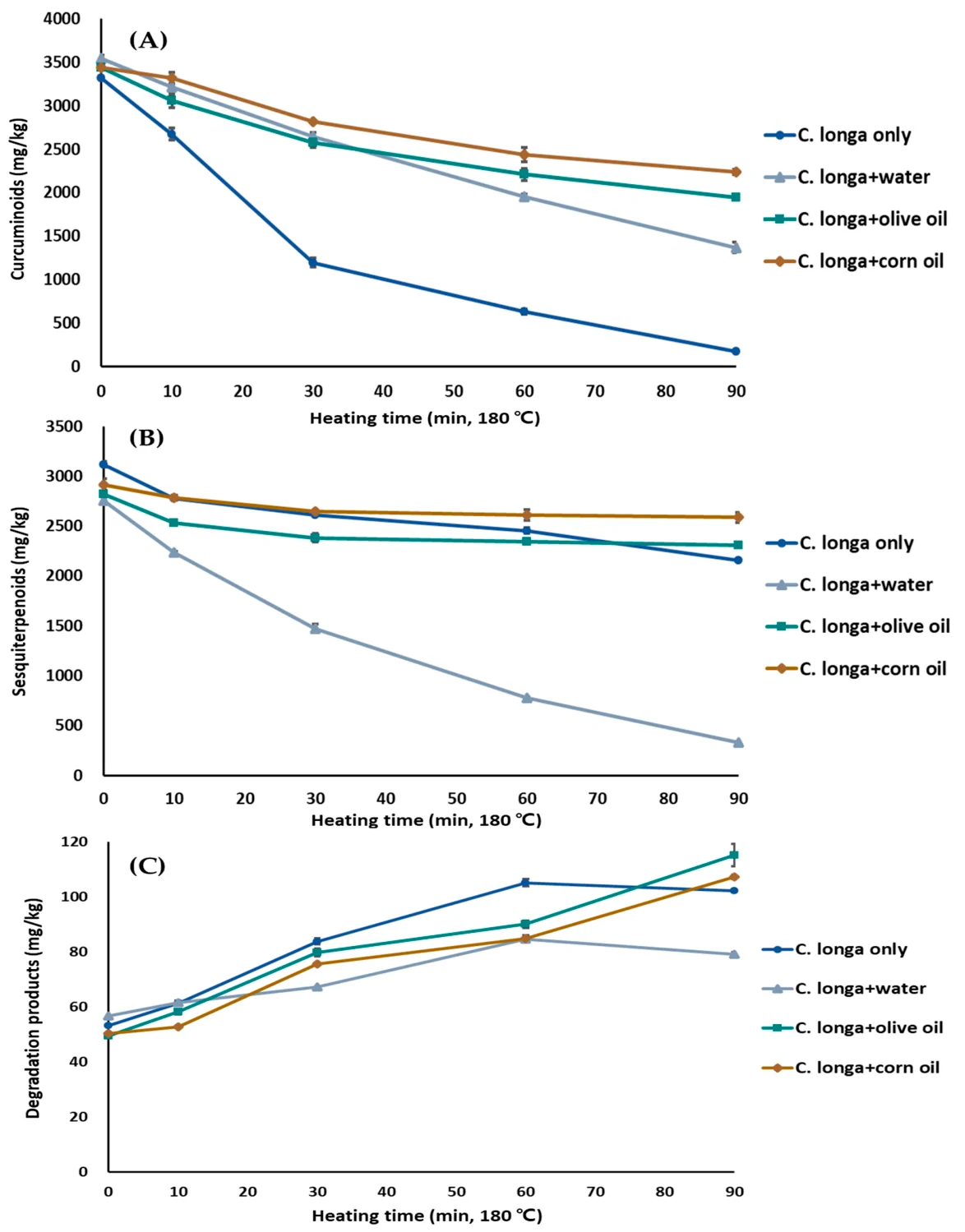
Matrix-dependent changes in major phytochemicals of *Curcuma longa* during thermal treatment at 180 °C. (**A**) Total curcuminoids (curcumin, demethoxycurcumin, bisdemethoxycurcumin); (**B**) sum of *ar-*turmerone and bisacurone; (**C**) thermal degradation products (vanillin, dehydrozingerone) in methanolic extracts under four heating matrices: Dry (*C. longa* only), Aqueous (*C. longa* + water), Olive oil (*C. longa* + olive oil), and Corn oil (*C. longa* + corn oil). Samples were heated for 0 (control), 10, 30, 60, and 90 min. Values represent mean ± SD (*n* = 3). Statistical comparisons for the individual compounds are provided in Table S4.

Thermal treatment at 180 °C induced pronounced, matrix-dependent changes in the phytochemical profile of *C. longa* (Figure 1, Table S4). The chemical structures of the major compounds evaluated in this study are provided in Figure S1. Curcuminoids (curcumin, demethoxycurcumin, bisdemethoxycurcumin) decreased progressively in all matrices, with the most rapid depletion observed under dry heating; total curcuminoids decreased sharply within 30 min and were nearly depleted by 90 min. The pronounced loss under dry heating is consistent with the known thermal instability of curcuminoids and their susceptibility to degradation at elevated temperatures [2,7,11]. However, the specific degradation pathways responsible for these changes were not directly investigated in the present study.

In contrast, oil-based matrices (olive and corn oil) significantly enhanced curcuminoid retention. The greater retention observed in the oil matrices may be associated with the lipophilic nature of curcuminoids and differences in the physicochemical environment during heating [11]. However, oxygen availability and partition behavior were not directly measured, and their contributions to curcuminoid stability therefore cannot be established from the present data. The aqueous matrix exhibited intermediate stability. Previous studies have reported oxidative and autoxidative degradation of curcumin in aqueous systems, including the formation of bicyclopentadione products [7]; however, these products and the corresponding degradation pathways were not evaluated in the present study.

Sesquiterpenoids displayed superior thermal stability compared to curcuminoids, particularly in oil matrices, where levels decreased only moderately. Unlike the progressive decline of curcuminoids, the sum of *ar-*turmerone and bisacurone showed most of its decrease during the earlier stage of heating in the dry and oil-based matrices, followed by relatively smaller changes during prolonged heating, whereas a continued decline was observed in the aqueous matrix. This distinct temporal pattern indicates different thermal responses of curcuminoids and the measured sesquiterpenoids, which may be associated with their markedly different chemical structures and physicochemical properties. The greater retention of the measured sesquiterpenoids in oil matrices may likewise reflect matrix-dependent physicochemical effects, although the underlying mechanisms were not directly determined.

While the parent compounds declined, the selected curcuminoid degradation markers vanillin and dehydrozingerone accumulated during heating, with more pronounced accumulation in oil-based matrices than in aqueous conditions. These increases occurred concurrently with the loss of curcuminoids; however, the present data do not establish a quantitative conversion of curcuminoids into vanillin or dehydrozingerone. Importantly, vanillin and dehydrozingerone were evaluated as selected curcuminoid degradation markers and therefore are not expected to quantitatively correspond to the loss of *ar-*turmerone and bisacurone shown in Figure 1B. Other degradation products, including bicyclopentadione-related products previously reported for curcumin [7,29], were not quantified in this study. Therefore, vanillin and dehydrozingerone should be regarded as selected degradation markers rather than as products accounting for the overall loss of curcuminoids. Future studies systematically comparing oil matrices with different physicochemical and compositional properties may help clarify the factors governing sesquiterpenoid stability during thermal processing.

### 3.2. Semi-Quantitative Profiling of Essential Oil Components in Thermally Processed C. longa Extracts Using GC–MS

**Figure 2:**
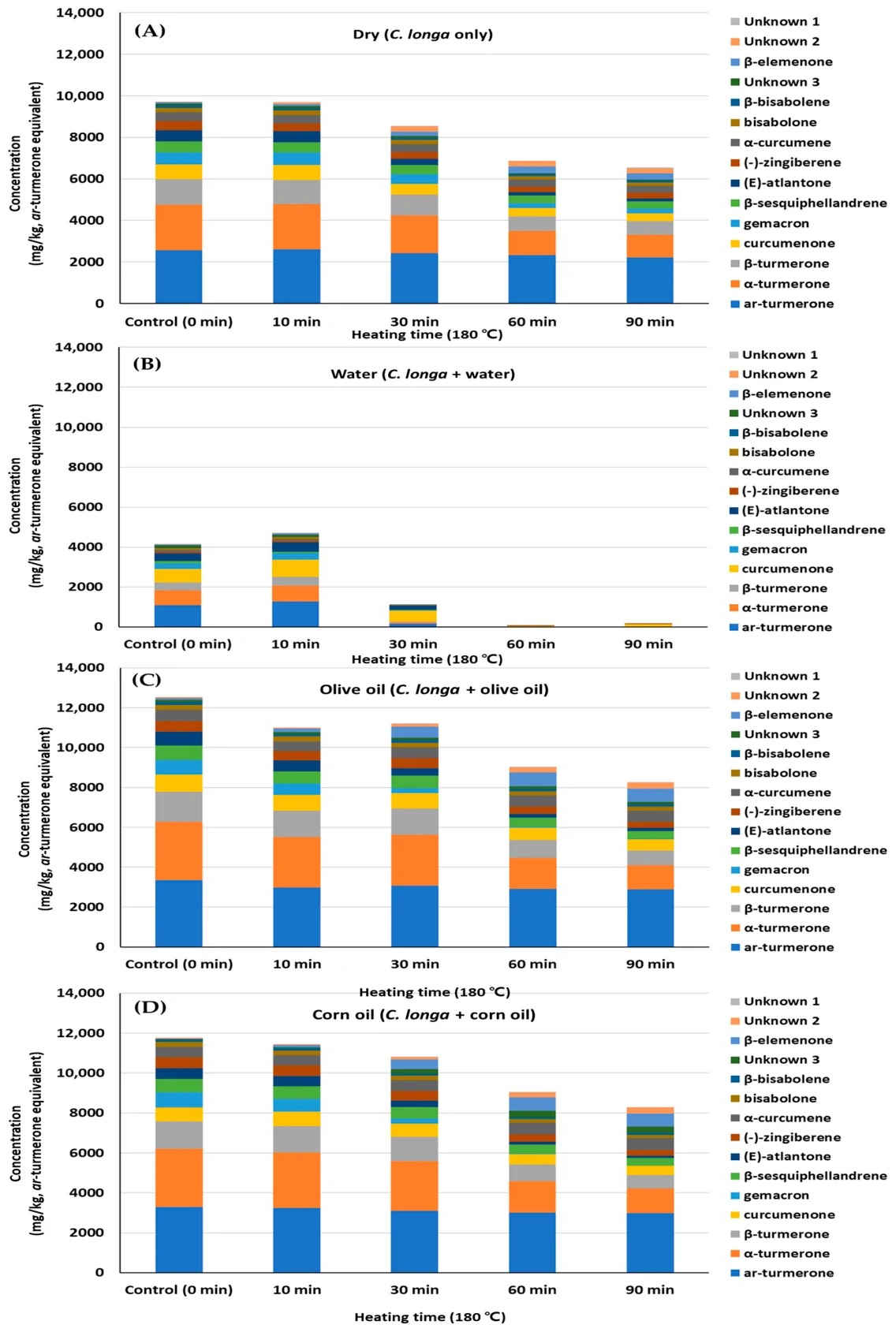
Changes in the composition of volatile constituents in hexane extracts of *Curcuma longa* during thermal treatment at 180 °C under four heating media: (**A**) Dry (*C. longa* only), (**B**) Aqueous (*C. longa* + water), (**C**) Olive oil (*C. longa* + olive oil), and (**D**) Corn oil (*C. longa* + corn oil) at 0 (control), 10, 30, 60, and 90 min. Relative contents of 15 major sesquiterpenes and other volatiles were quantified by GC–MS and expressed as *ar-*turmerone equivalents. Identified compounds included *ar-*turmerone, α-turmerone, β-turmerone, curcumenone, germacrone, and β-sesquiphellandrene; three additional peaks (Unknown 1–3) could not be structurally identified. Values represent mean (*n* = 3). The corresponding quantitative values and variability (mean ± SD, *n* = 3) for each compound are provided in Table S5.

Semi-quantitative GC–MS analysis, expressed as *ar-*turmerone equivalents, revealed distinct matrix-dependent alterations in the volatile profile of *C. longa* (Figure 2, Table S5).

Twelve predominant sesquiterpenes and related volatile compounds were identified, together with three unidentified peaks (U1–U3), with *ar-*turmerone and α-turmerone as the principal constituents across all matrices (Table S5). Although total essential oil content declined progressively at 180 °C, the extent of loss was highly dependent on the heating medium. The most pronounced depletion occurred in the aqueous matrix, where most volatiles were nearly eliminated after prolonged heating. This rapid loss may be associated with the volatility and hydrophobicity of sesquiterpenes and enhanced volatilization and mass transfer under aqueous heating conditions [5,30]. Conversely, the greater retention observed in the olive- and corn-oil matrices may be associated with the solubilization of these hydrophobic constituents in the lipid phase and reduced volatilization during heating [8,19]. However, comparison of the two oil matrices showed compound-dependent differences, and neither oil consistently provided greater retention across all volatile constituents.

The modest increases observed for some individual constituents between 10 and 30 min in the olive-oil matrix should also be interpreted within the semi-quantitative nature of the GC–MS analysis, as described in Section Semi-Quantitative Determination Using *ar*-Turmerone Equivalents.

Dry heating resulted in intermediate reductions, likely due to the gradual volatilization of low-boiling-point sesquiterpenes. Despite the overall decline, turmerone-type compounds remained relatively stable, particularly compared to more volatile minor constituents, consistent with reports of their inherent thermal resilience [1,5]. These matrix-mediated compositional shifts are expected to influence the antioxidant activity and lipid-protective performance of the extracts, given the radical-scavenging properties of turmerones [5,8]. Systematic comparison of oils with different fatty acid compositions could further elucidate how the characteristics of the heating oil influence the stability of volatile constituents during thermal processing.

### 3.3. Changes in Antioxidant Capacity of C. longa Extracts as Influenced by Thermal Processing and Matrix Conditions

**Figure 3:**
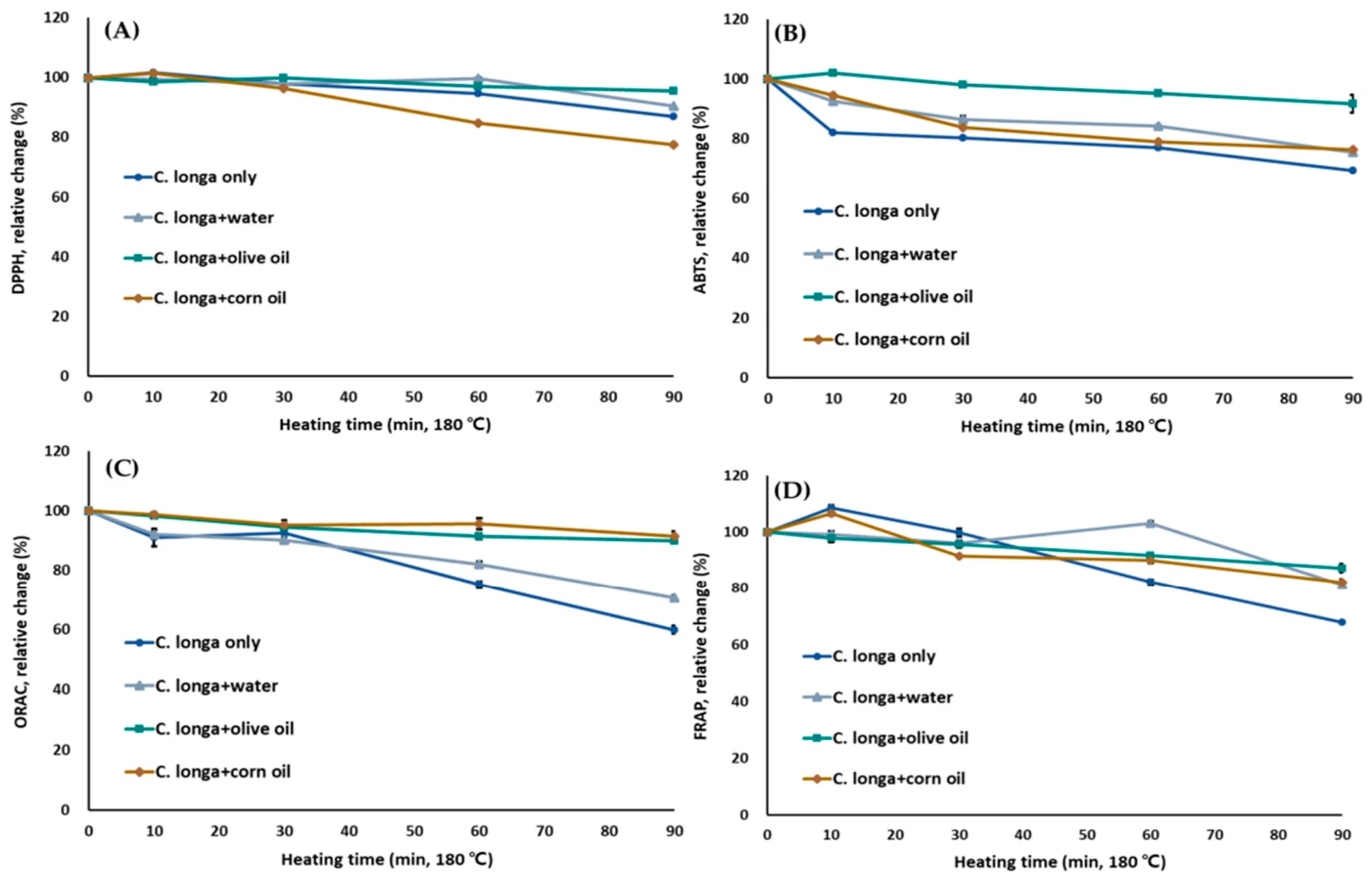
Relative changes (%) in antioxidant activities of *Curcuma longa* extracts following thermal treatment at 180 °C under different matrices. (**A**) DPPH radical-scavenging activity, (**B**) ABTS radical-scavenging activity, (**C**) oxygen radical absorbance capacity (ORAC), (**D**) ferric reducing antioxidant power (FRAP). Samples were heated under four matrices: Dry (*C. longa* only), Aqueous (*C. longa* + water), Olive oil (*C. longa* + olive oil), Corn oil (*C. longa* + corn oil) for 0 (control), 10, 30, 60, and 90 min. Values are expressed as percentages relative to the untreated control (100%). Values represent mean ± SD (*n* = 3).

Thermal processing led to a progressive decline in the antioxidant capacity of *C. longa* extracts, with the extent of loss strongly dependent on the matrix (Figure 3, Table 1). The greatest reductions in all antioxidant indices (ABTS, ORAC, DPPH, FRAP) were observed in the aqueous system, where prolonged heating resulted in approximately 24.5% and 29.4% decreases in ABTS and ORAC values, respectively, after 90 min.

At 90 min, the differences among heating matrices varied depending on the antioxidant assay (Table 1). Oil-based matrices generally showed greater retention of antioxidant capacity, although the magnitude and statistical significance of these differences were assay-dependent. This assay-dependent response may reflect differences in the reaction principles of DPPH, ABTS, ORAC, and FRAP and their differential responsiveness to the phytochemical composition retained after thermal processing. These trends were broadly consistent with the matrix-dependent changes in bioactive compounds described in Section 3.1, suggesting an association between phytochemical retention and antioxidant capacity during thermal processing.

TPC and TFC generally decreased during thermal processing, but their matrix-dependent changes did not necessarily parallel those of the individual antioxidant indices. This indicates that the antioxidant capacity of the extracts cannot be explained by TPC or TFC alone, but rather reflects the combined responses of multiple constituents measured differently by each antioxidant assay.

Notably, at 90 min, TFC was lower in the corn-oil treatment than in the aqueous treatment, despite greater retention of several individual phytochemicals in the oil-based matrices. This discrepancy may partly reflect the structure-dependent response of the AlCl_3_-based TFC assay [31], as well as differences in the composition of compounds retained or transformed during heating. Moreover, the antioxidant responses of the corn-oil and aqueous treatments at 90 min differed among DPPH, ABTS, ORAC, and FRAP, indicating that TFC alone did not account for the overall antioxidant response.

Taken together, these results indicate that lipid-based matrices generally reduced heat-induced losses of antioxidant activity, although the extent of retention was assay-dependent. The findings underscore the importance of matrix selection in food formulation and processing to maximize the functional quality of turmeric-derived products.

### 3.4. Associations Between Phytochemical Composition and Antioxidant Properties in Thermally Treated C. longa Extracts

Associations between phytochemical composition and antioxidant activities in thermally treated *C. longa* extracts are summarized in Table 2. Curcuminoids exhibited strong positive correlations with antioxidant assays, especially ORAC (r = 0.71–0.90), ABTS (r = 0.80–0.83), and FRAP (r = 0.75–0.84), indicating that changes in curcuminoid levels were closely associated with changes in antioxidant capacity during thermal processing. TPC also correlated strongly with ORAC (r = 0.76) and FRAP (r = 0.87), showing a similar association between phenolic content and antioxidant activity.

By contrast, degradation products such as vanillin and dehydrozingerone exhibited significant negative correlations with antioxidant indices (e.g., vanillin: ORAC, r = −0.60; ABTS, r = −0.68; FRAP, r = −0.68), indicating an inverse association between their accumulation and antioxidant capacity during heating.

Sesquiterpenoids displayed weaker correlations with antioxidant capacity. For example, *ar-*turmerone correlated moderately with ORAC (r = 0.51) and showed little association with TPC (r = 0.06), indicating weaker associations with the measured antioxidant indices than those observed for curcuminoids. These findings are consistent with previous reports describing the antioxidant properties of curcuminoids and essential oil components in turmeric [2,6].

Overall, curcuminoid and phenolic contents showed the strongest positive associations with antioxidant capacity in thermally processed *C. longa* (Ulgeum). However, these correlations should not be interpreted as evidence of a direct causal contribution, because curcuminoid levels and antioxidant activities both declined with heating time, and their common time-dependent changes may contribute to the observed correlations. Further quantitative characterization of additional phytochemical classes, including sesquiterpenoid and volatile constituents, will be necessary to evaluate their potential contributions to the antioxidant responses observed during thermal processing.

### 3.5. Influence of Thermally Processed C. longa Extracts on Lipid Oxidative Stability in Oil-in-Water (O/W) Emulsion Systems

To determine the functional impact of thermal processing on lipid oxidative stability, a standardized O/W emulsion model was employed. Given that matrix-dependent phytochemical stability was addressed in Section 3.1, Section 3.2 and Section 3.3, only extracts from dry thermal treatment were evaluated to specifically assess the effect of heat-induced compositional changes in *C. longa*. This approach also mirrors practical application, as turmeric extracts are typically incorporated into processed food systems rather than bulk oils, and the O/W emulsion model—with its large interfacial area—provides a sensitive platform for discriminating oxidative stability among treatments. However, because only dry-heated extracts were evaluated and emulsion pH, droplet size, polydispersity, and physical stability were not independently characterized, the oxidative-stability findings should be interpreted specifically within this experimental system and should not be generalized to extracts heated in aqueous, olive oil, or corn oil matrices.

#### 3.5.1. Headspace Oxygen Depletion

**Figure 4:**
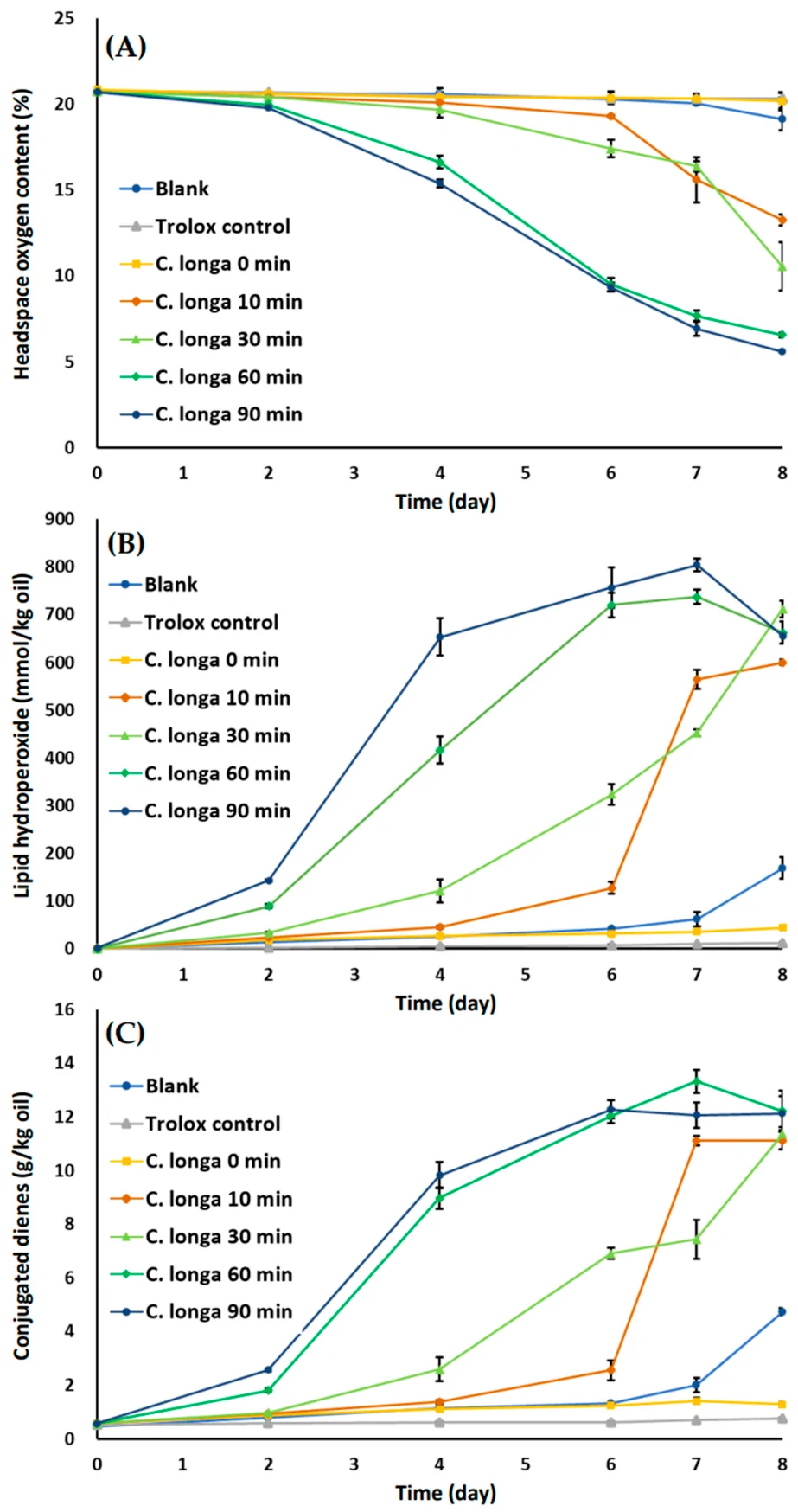
Oxidative stability of oil-in-water emulsions containing methanolic *Curcuma longa* extracts after thermal treatment at 180 °C. Changes in (**A**) headspace oxygen content, (**B**) lipid hydroperoxide formation, and (**C**) conjugated diene levels during storage at 25 °C for 8 days. Extracts from samples heated for 0 (control), 10, 30, 60, and 90 min were incorporated into the emulsion system. Blank (methanol) and Trolox control (adjusted to the same antioxidant capacity based on ORAC values) were included for comparison. Values represent mean ± SD (*n* = 3). Two-way ANOVA showed significant effects of treatment, storage time, and their interaction on all three oxidation indices (all *p* < 0.001).

Headspace oxygen content decreased during storage in O/W emulsion systems containing thermally processed *C. longa* extracts (Figure 4A). In contrast, the blank and Trolox control maintained oxygen levels near the initial value (~20%) over 8 days, as did the unheated extract (0 min; 20.17% on day 8). Thermally treated extracts exhibited a clear, time-dependent reduction in headspace oxygen: after 8 days, levels decreased to 13.25%, 10.54%, 6.56%, and 5.60% for the 10-, 30-, 60-, and 90-min treatments, respectively. The extent and rate of oxygen depletion increased with heating duration: in the 10- and 30-min samples, oxygen decreased gradually, whereas in the 60- and 90-min treatments, a pronounced drop occurred after day 4. Two-way ANOVA confirmed significant effects of treatment, storage time, and their interaction on headspace oxygen levels (all *p* < 0.001).

These findings indicate that thermal processing alters the oxygen consumption kinetics of *C. longa* extracts in emulsion systems. Because oxygen is continuously consumed during the propagation stage of lipid oxidation, the accelerated oxygen depletion observed after prolonged heating suggests that thermally processed extracts promoted oxidative chain reactions rather than simply scavenging dissolved oxygen. In oil-in-water emulsions, oxidative stability depends not only on the intrinsic antioxidant activity of phytochemicals but also on their partitioning between the oil, aqueous, and interfacial regions, which governs their accessibility to lipid radicals. Therefore, thermal degradation may have altered the distribution of phytochemicals within the emulsion, resulting in enhanced oxygen consumption despite the retention of measurable antioxidant capacity [12,13,32].

#### 3.5.2. Lipid Hydroperoxide Formation

Lipid hydroperoxide values increased during storage in all emulsion systems; however, the magnitude and rate of accumulation differed markedly among treatments (Figure 4B). The blank exhibited a rapid rise, reaching 169.43 mmol/kg oil by day 8. Because the blank contained the same oxidizable lipid phase and was subjected to the same accelerated storage conditions, this increase represents background lipid oxidation occurring in the emulsion system in the absence of *C. longa* extract. In contrast, the Trolox control suppressed oxidation, maintaining low hydroperoxide values (reaching 11.68 mmol/kg oil at day 8). The unheated *C. longa* extract (0 min) provided moderate protection, with hydroperoxide values of 44.41 mmol/kg oil at day 8.

Thermally processed extracts resulted in substantially higher hydroperoxide formation, with values after 8 days reaching 600.29, 712.19, 662.57, and 655.33 mmol/kg oil for the 10-, 30-, 60-, and 90-min treatments, respectively. The temporal patterns also varied: the 10- and 30-min samples showed accelerated hydroperoxide formation after day 4, while the 60- and 90-min treatments exhibited rapid increases from the early stages of storage. The highest value was observed in the 90-min treatment at day 7 (804.39 mmol/kg oil). Two-way ANOVA confirmed significant effects of treatment, storage time, and their interaction on lipid hydroperoxide formation (all *p* < 0.001).

These results demonstrate that thermal processing of *C. longa* extracts markedly enhances lipid hydroperoxide formation in O/W emulsions. Lipid hydroperoxides are the primary products generated during the initiation and propagation stages of lipid oxidation; therefore, their rapid accumulation indicates that thermal treatment shifted the functional behavior of the extracts toward promoting oxidative chain reactions. The substantial increase in hydroperoxide formation also suggests that changes in phytochemical composition during heating exerted a greater influence on oxidation than the antioxidant activity measured in homogeneous chemical assays [12,13,32,33].

#### 3.5.3. Conjugated Diene (CD) Development

CD values increased during storage in all emulsion systems, with distinct differences among treatments (Figure 4C). The blank exhibited a steady rise, reaching 4.74 g/kg oil by day 8, whereas the Trolox control remained low throughout (<0.76 g/kg oil). The unheated *C. longa* extract (0 min) resulted in limited CD formation (1.28 g/kg oil at day 8).

Thermally processed extracts exhibited substantially higher CD levels: after 8 days, values reached 11.12, 11.35, 12.22, and 12.13 g/kg oil for the 10-, 30-, 60-, and 90-min treatments, respectively. Temporal patterns differed: the 60- and 90-min treatments showed rapid increases from day 2, reaching ~9–10 g/kg oil by day 4, whereas the 10- and 30-min treatments increased more gradually until day 6, followed by sharper rises. Two-way ANOVA confirmed significant effects of treatment, storage time, and their interaction on CD formation (all *p* < 0.001).

Overall, thermally processed extracts showed greater CD formation than the unheated extract. During free-radical oxidation of polyunsaturated fatty acids, hydrogen abstraction from a bis-allylic position generates a resonance-stabilized carbon-centered radical, accompanied by rearrangement of the double-bond system to form a conjugated diene structure. Subsequent reaction of the carbon-centered radical with molecular oxygen produces lipid peroxyl radicals and ultimately lipid hydroperoxides [34,35]. Thus, conjugated diene development and hydroperoxide formation are closely associated manifestations of primary lipid oxidation rather than strictly sequential events. Their accumulation together with increasing hydroperoxide levels confirms that thermal processing accelerated primary lipid oxidation in the emulsion system. However, the present CD assay quantified total conjugated dienes and did not distinguish individual positional or geometric isomers; therefore, the relative formation of different cis/trans diene configurations cannot be determined from the present data. Because susceptibility to bis-allylic hydrogen abstraction and subsequent oxidation depends on fatty acid structure, future studies incorporating detailed fatty acid profiling and characterization of conjugated diene isomers could provide further mechanistic insight into lipid oxidation in this emulsion system. These observations further indicate that the oxidative behavior of thermally processed *C. longa* extracts was governed by their localization within the multiphase system rather than solely by their radical-scavenging capacity, emphasizing the importance of interfacial phenomena in determining antioxidant performance in emulsions [12,13,32].

#### 3.5.4. Effect of EDTA on Oxidative Stability and Potential Metal-Mediated Oxidation

The impact of thermally processed *C. longa* extracts on lipid oxidative stability was evaluated in an O/W emulsion model system, which provides a model relevant to many multiphase food systems. In these systems, oxidation predominantly occurs at the oil–water interface, where lipids, oxygen, transition metals, and antioxidant compounds interact [10,12]. The interfacial localization of phenolic antioxidants is particularly important because their distribution among the oil, aqueous, and interfacial regions can substantially influence their effectiveness against lipid oxidation [36]. Continuous white LED illumination (14,600 lux) was applied throughout storage as the illumination condition.

Despite relatively preserved antioxidant capacity observed in chemical assays (Section 3.3), thermally processed extracts—especially after prolonged heating (60–90 min)—showed greater lipid oxidation in the emulsion system, as evidenced by rapid headspace oxygen depletion, increased hydroperoxides, and elevated CD values. This apparent discrepancy between chemical antioxidant assays and emulsion oxidation may reflect differences in the physicochemical environment, because antioxidant effectiveness in O/W emulsions depends not only on intrinsic radical-scavenging activity but also on the localization of antioxidant compounds and their interactions with prooxidants such as transition metals [12,13,36,37].

Thermal processing was accompanied by substantial changes in the phytochemical composition of the extracts, including losses of curcuminoids and increased levels of smaller phenolic compounds such as vanillin and dehydrozingerone. However, the specific contributions of these individual compounds to lipid oxidation cannot be inferred from their concentrations alone. The oxidative behavior of phenolic compounds in lipid systems can vary with the physicochemical environment and the nature of the prooxidant [37,38]. Therefore, the enhanced oxidation observed after thermal processing may reflect changes in the overall composition and physicochemical behavior of the extracts rather than the action of a specific degradation product.

Previous studies have shown that the oxidative behavior of curcumin can depend on matrix type and visible-light exposure [39], and its photosensitizing properties and singlet-oxygen generation under irradiation have also been reported in model systems [40]. However, photosensitized lipid oxidation can proceed through both Type I and Type II pathways. Type I reactions involve electron or hydrogen transfer between an excited photosensitizer and molecular oxygen or lipids, resulting in radical species, whereas Type II reactions involve energy transfer from the excited photosensitizer to molecular oxygen to generate singlet oxygen [41]. Therefore, lipid hydroperoxide formation under the present illumination conditions cannot by itself establish singlet oxygen as the initiating reactive species. Because a dark-storage control, singlet-oxygen quenching experiment, and structural characterization of diagnostic lipid oxidation products were not included in the present study, the relative contributions of Type I and Type II pathways cannot be determined. Future mechanistic studies using controlled singlet-oxygen generation with an endoperoxide, together with characterization of diagnostic oxidation products, would provide a direct approach for comparing singlet-oxygen-derived oxidation with the oxidation observed in the present illuminated emulsion system.

**Figure 5:**
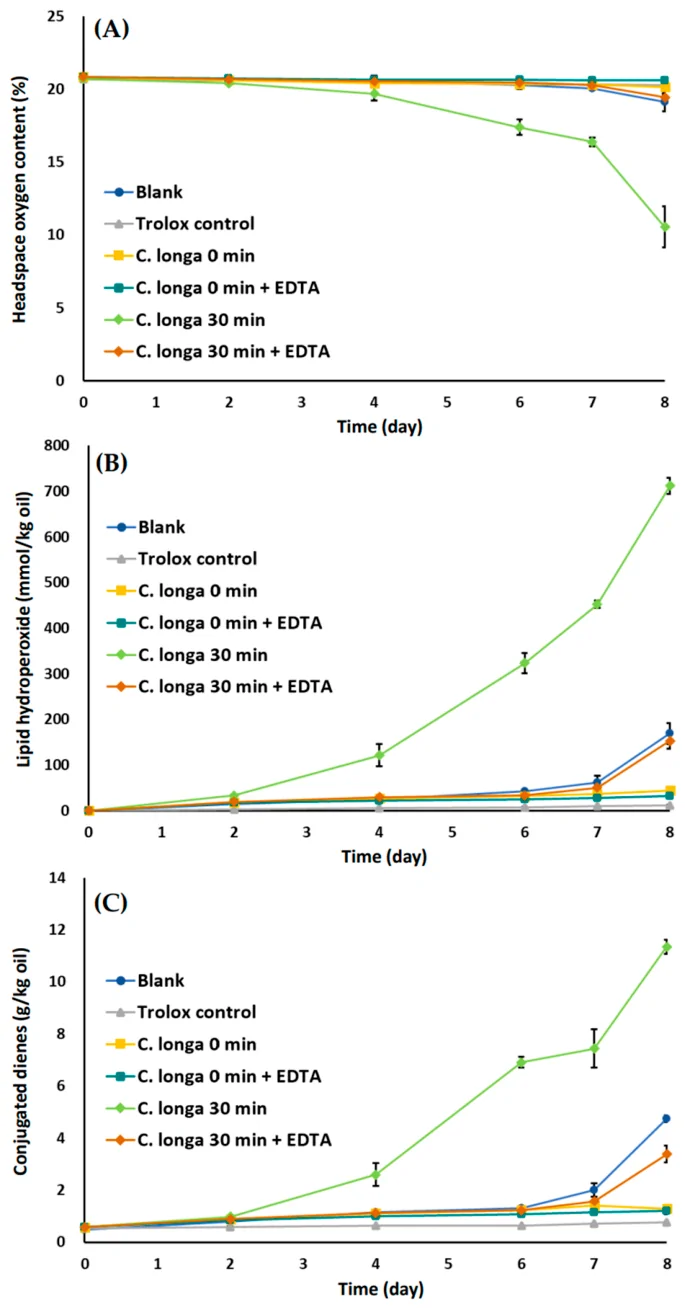
Effect of EDTA addition on oxidative stability of oil-in-water emulsions containing methanolic *Curcuma longa* extracts heated at 180 °C. Extracts obtained after 0 and 30 min of dry thermal treatment were evaluated to assess the potential prooxidant role of metal ions released during heating. Changes in (**A**) headspace oxygen content, (**B**) lipid hydroperoxide formation, and (**C**) conjugated diene levels were monitored during storage at 25 °C for 8 days. EDTA was added as a metal chelator to assess metal ion-mediated oxidation. Blank (methanol) and Trolox control were included for comparison. Values represent mean ± SD (*n* = 3). Two-way ANOVA showed significant effects of treatment, storage time, and their interaction on all three oxidation indices (all *p* < 0.001).

The potential involvement of metal-mediated oxidation was further examined by EDTA addition (Figure 5). EDTA markedly suppressed headspace oxygen depletion (Figure 5A), lipid hydroperoxide formation (Figure 5B), and conjugated diene accumulation (Figure 5C), particularly in extracts heated for 30 min. Previous studies have demonstrated that iron localization within emulsion systems can promote lipid oxidation and that EDTA can inhibit oxidation by altering the availability and interactions of iron within the emulsion [42]. Notably, the pronounced prooxidant behavior observed after thermal processing was strongly attenuated by EDTA, whereas the unheated extract showed substantially less oxidative activity. However, the EDTA experiment does not establish the source or chemical form of the metals involved, and further metal-speciation or fractionation studies would be required to determine whether thermal processing alters their chemical environment or availability. Nevertheless, the marked suppression of heat-associated oxidation by EDTA, together with the substantially lower oxidative activity of the unheated extract, supports the potential involvement of EDTA-sensitive, metal-mediated reactions in the altered oxidative behavior of thermally processed *C. longa* extracts.

These results align with previous research indicating that curcumin’s antioxidant or prooxidant behavior is matrix-dependent [39] and that partitioning and interfacial localization are critical determinants of oxidative stability in multiphase systems. Overall, thermal processing induces compositional changes in *C. longa* extracts that differentially affect antioxidant functionality depending on the reaction matrix. Although antioxidant capacity may appear retained in simplified assays, these extracts can promote lipid oxidation in complex systems through photo-oxidative, metal-mediated, and interfacial mechanisms. This underscores the importance of evaluating bioactive compounds under application-relevant conditions to accurately predict their functionality in the tested accelerated emulsion model.

## 4. Conclusions

Thermal processing of *C. longa* at 180 °C induced pronounced matrix-dependent changes in phytochemical composition and antioxidant properties. Curcuminoids progressively decreased with increasing heating time, whereas degradation products such as vanillin and dehydrozingerone accumulated, resulting in substantial compositional shifts. Oil-based matrices most effectively preserved curcuminoids and generally showed greater retention of antioxidant capacity, although the extent varied among antioxidant assays, whereas greater degradation occurred under aqueous and dry heating conditions.

Although antioxidant capacity was partially retained in chemical assays, oil-in-water emulsion studies under continuous LED illumination demonstrated that thermally processed extracts, particularly after prolonged heating, accelerated lipid oxidation, as evidenced by increased oxygen consumption, lipid hydroperoxide formation, and conjugated diene accumulation. The marked inhibitory effect of EDTA supported the potential involvement of EDTA-sensitive, metal-mediated reactions, while the contribution of photosensitization under the applied illumination conditions could not be directly established.

These findings demonstrate that antioxidant capacity measured in homogeneous chemical assays does not necessarily predict oxidative behavior in complex multiphase systems. Thermal processing modifies phytochemical composition and can consequently alter the balance between antioxidant and prooxidant behavior depending on the reaction environment.

Overall, this study provides insight into how heating matrices influence phytochemical stability, antioxidant properties, and oxidative behavior in *C. longa*. The limitations of this study include the use of a single turmeric source and heating temperature, the absence of a dark-storage control and direct characterization of metal speciation, and the evaluation of only primary lipid oxidation products. These findings highlight the importance of evaluating phytochemical functionality in multiphase model systems and provide a scientific basis for further investigation of thermally processed turmeric-derived ingredients.

## Figures and Tables

**Table 1:** Effects of thermal treatment at 180 °C under different heating conditions on antioxidant activities, total phenolic content (TPC), and total flavonoid content (TFC) of *Curcuma longa* extracts.

Heating Condition	Heating Time	TPC mmol GAE/kg	TFC mmol CE/kg	DPPH mmol TE/kg	ABTS mmol TE/kg	ORAC mmol TE/kg	FRAP mmol FeSO_4_ Eq/kg
Dry (*C. longa* only)	0 min (control)	58.43 ± 0.94 a	22.55 ± 0.37 a	21.39 ± 0.07 a	99.57 ± 0.36 a	260.57 ± 6.32 a	60.97 ± 0.51 b
	10 min	58.79 ± 1.21 a	19.79 ± 0.56 b	21.73 ± 0.09 a	81.66 ± 0.36 b	237.22 ± 7.88 b	66.25 ± 0.63 a
	30 min	55.84 ± 1.21 b	18.65 ± 0.37 c	20.93 ± 0.05 b	79.90 ± 0.28 c	241.20 ± 3.82 b	60.87 ± 0.88 b
	60 min	44.23 ± 0.68 c	10.95 ± 0.19 d	20.25 ± 0.08 c	76.59 ± 0.40 d	195.90 ± 2.85 c	50.15 ± 0.51 c
	90 min	35.23 ± 0.94 dD	10.07 ± 0.19 eD	18.60 ± 0.03 dC	69.14 ± 0.32 eC	156.12 ± 3.40 dD	41.30 ± 0.26 dC
Water (*C. longa* + water)	0 min (control)	64.32 ± 1.11 a	22.72 ± 0.75 a	21.49 ± 0.05 a	99.49 ± 0.43 a	266.77 ± 10.60 a	68.78 ± 0.76 b
	10 min	63.65 ± 0.62 a	21.73 ± 0.93 a	21.35 ± 0.13 a	92.11 ± 0.43 b	245.70 ± 4.14 b	68.20 ± 0.88 b
	30 min	62.76 ± 0.89 a	20.18 ± 0.37 b	21.05 ± 0.06 b	86.12 ± 1.08 c	240.54 ± 2.19 b	66.15 ± 0.63 c
	60 min	61.48 ± 0.89 a	18.86 ± 0.56 b	21.43 ± 0.05 a	83.79 ± 0.36 d	219.03 ± 2.50 c	70.96 ± 0.51 a
	90 min	45.86 ± 1.11 bC	14.68 ± 0.37 cB	19.41 ± 0.02 cB	75.10 ± 0.38 eB	188.21 ± 2.06 dC	55.80 ± 0.63 dB
Olive oil (*C. longa* + olive oil)	0 min (control)	58.73 ± 0.91 a	22.79 ± 0.93 a	21.56 ± 0.09 a	97.41 ± 0.46 a	267.80 ± 3.35 a	70.13 ± 0.63 a
	10 min	56.00 ± 0.91 ab	20.76 ± 0.75 b	21.27 ± 0.04 a	99.38 ± 0.36 a	263.06 ± 1.78 a	68.65 ± 1.01 a
	30 min	55.60 ± 1.73 b	18.13 ± 0.75 c	21.52 ± 0.14 a	95.62 ± 0.70 b	253.04 ± 6.31 b	67.05 ± 0.76 b
	60 min	54.49 ± 0.65 c	17.63 ± 0.38 c	20.94 ± 0.09 b	92.66 ± 0.44 c	245.04 ± 2.87 b	64.28 ± 0.76 c
	90 min	52.51 ± 1.18 dA	15.95 ± 0.56 dA	20.57 ± 0.04 cA	89.26 ± 2.89 dA	240.89 ± 0.44 cA	61.19 ± 1.01 dA
Corn oil (*C. longa* + corn oil)	0 min (control)	58.50 ± 0.87 a	23.01 ± 0.75 a	21.14 ± 0.05 a	100.41 ± 1.20 a	262.55 ± 0.23 a	70.60 ± 0.89 a
	10 min	57.34 ± 0.86 a	20.96 ± 0.94 b	21.43 ± 0.04 a	94.86 ± 0.72 b	259.32 ± 1.70 a	75.33 ± 0.51 a
	30 min	52.46 ± 1.68 b	18.28 ± 0.37 c	20.37 ± 0.05 b	84.27 ± 0.23 c	249.85 ± 4.74 b	64.66 ± 0.25 b
	60 min	51.89 ± 1.68 b	17.50 ± 0.56 c	17.89 ± 0.04 c	79.38 ± 0.38 d	251.01 ± 4.82 b	63.58 ± 0.76 c
	90 min	47.50 ± 0.72 cB	12.95 ± 0.56 dC	16.37 ± 0.05 dD	76.64 ± 0.13 eB	240.23 ± 4.20 cB	57.90 ± 0.88 dB

Notes: 1. Values represent mean ± SD (*n* = 3). Two-way ANOVA was used to evaluate the effects of heating matrix, heating time, and their interaction. 2. Within each matrix, different lowercase letters in the same column denote significant differences among heating times according to Tukey’s post hoc test (*p* < 0.05). Uppercase letters in the 90 min row indicate significant differences among matrices in the percentage reduction from 0 to 90 min according to Tukey’s post hoc test (*p* < 0.05), with A representing the smallest percentage reduction. 3. Antioxidant activities determined by 2,2-diphenyl-1-picrylhydrazyl (DPPH), 2,2′-azinobis(3-ethylbenzothiazoline-6-sulfonic acid) (ABTS), and oxygen radical absorbance capacity (ORAC) are expressed as millimoles Trolox equivalents per kilogram. Ferric reducing antioxidant power (FRAP) is expressed as millimoles FeSO_4_ equivalents per kilogram. Total phenolic content (TPC) and total flavonoid content (TFC) are expressed as millimoles gallic acid equivalents per kilogram and millimoles catechin equivalents per kilogram, respectively.

**Table 2:** Pearson correlation analysis linking bioactive compounds, phenolic and flavonoid contents, and antioxidant activities in thermally treated *Curcuma longa* extracts.

	DMC	BDMC	BS	AT	VNL	DHG	TFC	TPC	DPPH	ABTS	ORAC	FRAP
**CUR**	0.97 ***	0.86 ***	0.76 ***	0.37	−0.73 ***	−0.75 ***	0.86 ***	0.77 ***	0.41	0.83 ***	0.90 ***	0.84 ***
**DMC**		0.96 ***	0.79 ***	0.31	−0.86 ***	−0.87 ***	0.92 ***	0.81 ***	0.51 *	0.83 ***	0.84 ***	0.83 ***
**BDMC**			0.78 ***	0.23	−0.92 ***	−0.95 ***	0.92 ***	0.80 ***	0.60 **	0.80 ***	0.71 ***	0.75 ***
**BS**				0.54 *	−0.74 ***	−0.72 ***	0.80 ***	0.64 **	0.51 *	0.73 ***	0.78 ***	0.58 **
**AT**					−0.18	−0.23	0.23	0.06	0.05	0.37	0.51 *	0.10
**VNL**						0.98 ***	−0.87 ***	−0.67 **	−0.62 **	−0.68 **	−0.60 **	−0.68 **
**DHG**							−0.87 ***	−0.68 **	−0.59 **	−0.69 **	−0.58 **	−0.66 **
**TFC**								0.90 ***	0.69 **	0.83 ***	0.82 ***	0.85 ***
**TPC**									0.69 **	0.71 ***	0.76 ***	0.87 ***
**DPPH**										0.67 **	0.41	0.59 **
**ABTS**											0.82 ***	0.74 ***
**ORAC**												0.82 ***

Notes: 1. Values represent Pearson correlation coefficients (r) calculated using the mean values of 20 experimental conditions (four heating matrices × five heating times; *n* = 20). 2. *p*-values were adjusted for multiple comparisons using the Benjamini–Hochberg false discovery rate (FDR) procedure. Statistical significance was indicated as * for FDR-adjusted *p* < 0.05, ** for FDR-adjusted *p* < 0.01, and *** for FDR-adjusted *p* < 0.001. 3. Abbreviations: CUR, curcumin; DMC, demethoxycurcumin; BDMC, bisdemethoxycurcumin; BS, bisacurone; AT, *ar-*turmerone; VNL, vanillin; DHG, dehydrozingerone; TPC, total phenolic content; TFC, total flavonoid content; DPPH, 2,2-diphenyl-1-picrylhydrazyl; ABTS, 2,2′-azinobis-(3-ethylbenzothiazoline-6-sulfonic acid); ORAC, oxygen radical absorbance capacity; FRAP, ferric reducing antioxidant power.

## Data Availability

The data supporting the findings of this study are included in the article and Supplementary Materials. Additional data retained by the authors are available from the corresponding author upon reasonable request.

## Supplementary Materials

The following supporting information can be downloaded at: https://www.mdpi.com/article/10.3390/antiox15081030/s1, Table S1: Optimized MRM transitions and instrumental parameters for UHPLC–MS/MS quantification of sesquiterpenoids and curcuminoids. Table S2: Optimized MRM transitions and instrumental parameters for UHPLC–MS/MS quantification of thermal degradation products. Table S3: Detailed UHPLC and MS/MS operating conditions for quantification of bioactive compounds in *Curcuma longa*. Table S4: Matrix-dependent changes in seven bioactive compounds of *Curcuma longa* (Ulgeum) extracts after heating at 180 °C. Table S5: Quantitative profiles of 15 predominant sesquiterpenes and related volatile compounds in *Curcuma longa* (Ulgeum) extracts after thermal exposure at 180 °C for various durations. Figure S1. Chemical structures of the major phytochemicals and selected thermal degradation markers investigated in *Curcuma longa*.
