# Supplementary material for: Heat-Induced Phytochemical Changes in Curcuma longa: Effects on Antioxidant Properties and Oxidative Behavior in Illuminated Oil-in-Water Emulsions
Source: Antioxidants (Basel). 2026 Aug 19;15(8):1030. doi: 10.3390/antiox15081030 (PMC13510205; doi:10.3390/antiox15081030)
Supplement: Supplementary file 1 [file antioxidants-15-01030-s001.zip › antioxidants-4478972-supplementary.pdf]

Supporting Information for

Heat-induced phytochemical changes in *Curcuma longa*: Effects on antioxidant properties and oxidative behavior in illuminated oil-in-water emulsions

Choong-In Yun [1,2], Juhee Cho [3], Ji-Won Jeong [1], Young-Jun Kim [2,3*], JaeHwan Lee [1*]

[1] *Department of Food Science and Biotechnology, Sungkyunkwan University, Suwon 16419, Korea*

[2] *Research Institute of Food and Biotechnology, Seoul National University of Science and Technology, Seoul 01811, Korea*

[3] *Department of Food Science and Biotechnology, Seoul National University of Science and Technology, Seoul 01811, Korea*

Corresponding authors.

E-mail addresses: kimyj@seoultech.ac.kr (Y. Kim), s3hun@skku.edu (J. Lee).

Table S1. Optimized MRM transitions and instrumental parameters for UHPLC–MS/MS quantification of sesquiterpenoids and curcuminoids.

Compound	Ion mode	Precursor (*m/z*)	Quantifier (*m/z*, CE eV)	Qualifier 1 (*m/z*, CE eV)	Qualifier 2 (*m/z*, CE eV)	Dwell Time (ms)	RF Lens (V)	t_R (min)
Bisacurone	ESI+	235.2	217.1 (5)	119.0 (12)	91.0 (34)	50	59	3.30
BDMC	ESI+	309.1	147.0 (20)	119.0 (33)	-	50	114	3.84
DMC	ESI+	339.1	146.9 (23)	176.9 (16)	-	50	123	3.89
Curcumin	ESI+	369.1	145.0 (31)	176.9 (19)	-	50	124	3.93
ar-Turmerone	ESI+	217.2	91.0 (33)	117.0 (28)	-	50	73	4.44

Table S2. Optimized MRM transitions and instrumental parameters for UHPLC–MS/MS quantification of thermal degradation products.

Compound	Ion mode	Precursor (*m/z*)	Quantifier (*m/z*, CE eV)	Qualifier (*m/z*, CE eV)	Dwell Time (ms)	RF Lens (V)	t_R (min)
Vanillin	ESI−	151.0	135.9 (13)	108.0 (22)	100	85	5.30
Dehydrozingerone	ESI+	193.0	174.9 (13)	142.9 (17)	100	114	5.72
Vanillin- $^{13}C_6$ (IS)	ESI+	159.0	98.9 (15)	-	100	83	5.30

Table S3. Detailed UHPLC and MS/MS operating conditions for quantification of bioactive compounds in *Curcuma longa*.

(A) UHPLC conditions

Parameter	Curcuminoids & Sesquiterpenoids	Vanillin & Dehydrozingerone
UHPLC system	Vanquish Flex UHPLC (Thermo Fisher Scientific)	
Column	ZORBAX Eclipse Plus C18 (2.1 × 50 mm, 1.8 μm)	Imtakt Unison UK C18 (2.0 × 100 mm, 3 μm)
Column temperature	45 °C	30 °C
Mobile phase A	0.1% formic acid in water	0.1% formic acid in water
Mobile phase B	0.1% formic acid in acetonitrile	0.1% formic acid in acetonitrile
Flow rate	0.5 mL/min	0.2 mL/min
Injection volume	2 μL	5 μL
Gradient program	0–0.1 min, 5% B; 0.1–2.0 min, 5–25% B; 2.0–4.0 min, 25–95% B; 4.0–4.5 min, 95% B; 4.6–6.0 min re-equilibration	0–1 min, 10% B; 1–5 min, 10–90% B; 5–6 min, 90% B; 6.1–11 min re-equilibration

(B) MSMS conditions

Parameter	Setting
Mass spectrometer	TSQ Altis™ Plus Triple Quadrupole (Thermo Fisher Scientific)
Ionization source	Heated electrospray ionization (H-ESI)
Ionization mode	Positive (curcuminoids, sesquiterpenoids, dehydrozingerone); Negative (vanillin)
Spray voltage	3500 V (ESI^+); 3000 V (ESI^-)
Sheath gas	50 arbitrary units
Auxiliary gas	10 arbitrary units
Sweep gas	1 arbitrary unit
Vaporizer temperature	350 °C
Ion transfer tube temperature	325 °C

Table S4. Matrix-dependent changes in seven bioactive compounds of *Curcuma longa* (Ulgeum) extracts after heating at 180 °C.

Heating condition	Heating time	CUR (mg/kg)	DMC (mg/kg)	BDMC (mg/kg)	BS (mg/kg)	AT (mg/kg)	VNL (mg/kg)	DHG (mg/kg)
Dry (*C. longa* only)	0 min (control)	2493.8 ± 18.4 a	590.4 ± 3.6 a	233.7 ± 1.5 a	474.9 ± 1.8 a	2642.2 ± 27.4 a	46.7 ± 0.3 e	6.4 ± 0.0 e
	10 min	2003.3 ± 56.5 b	481.9 ± 21.0 b	185.5 ± 3.7 b	323.0 ± 10.7 b	2450.7 ± 9.5 b	51.4 ± 0.2 d	10.0 ± 0.2 d
	30 min	913.5 ± 41.5 c	204.3 ± 9.2 c	78.3 ± 1.4 c	264.1 ± 5.9 c	2345.1 ± 5.8 c	64.8 ± 1.1 c	19.0 ± 0.0 c
	60 min	474.2 ± 24.2 d	110.0 ± 4.9 d	49.2 ± 2.1 d	194.7 ± 1.4 d	2258.1 ± 30.8 d	80.9 ± 0.9 b	24.1 ± 0.5 b
	90 min	121.1 ± 1.9 eD	33.4 ± 0.5 eD	20.1 ± 0.5 eD	85.4 ± 1.6 eD	2070.8 ± 13.9 eC	80.6 ± 0.4 aC	21.5 ± 0.3 aC
Water (*C. longa* + water)	0 min (control)	2652.3 ± 26.6 a	635.4 ± 9.1 a	251.8 ± 4.6 a	296.4 ± 10.7 a	2460.0 ± 4.1 a	49.8 ± 0.2 e	7.0 ± 0.1 e
	10 min	2412.8 ± 7.1 b	570.8 ± 1.2 b	225.6 ± 3.0 b	251.3 ± 9.9 b	1980.2 ± 14.3 b	53.5 ± 0.8 d	8.1 ± 0.1 d
	30 min	1978.1 ± 36.3 c	482.3 ± 17.0 c	187.0 ± 6.3 c	240.8 ± 5.7 c	1232.0 ± 50.3 c	56.7 ± 0.1 c	10.5 ± 0.1 c
	60 min	1464.0 ± 31.4 d	348.5 ± 4.8 d	136.7 ± 2.6 d	208.9 ± 8.7 d	566.5 ± 17.6 d	67.6 ± 0.1 b	17.1 ± 0.1 b
	90 min	1027.5 ± 50.6 eC	243.7 ± 9.3 eC	99.6 ± 4.2 eC	134.9 ± 6.1 eC	194.9 ± 6.9 eD	61.4 ± 0.8 aD	17.7 ± 0.0 aD
Olive oil (*C. longa* + olive oil)	0 min (control)	2586.5 ± 15.1 a	615.0 ± 3.8 a	240.0 ± 1.5 a	347.6 ± 15.8 a	2469.7 ± 30.5 a	43.8 ± 0.4 e	5.7 ± 0.3 e
	10 min	2294.6 ± 60.6 b	552.6 ± 27.8 b	207.8 ± 4.1 b	283.7 ± 8.7 b	2248.0 ± 0.1 b	50.2 ± 1.0 d	8.1 ± 0.0 d
	30 min	2028.0 ± 44.0 c	417.6 ± 6.6 c	125.1 ± 1.1 c	262.1 ± 5.2 c	2119.0 ± 48.4 c	62.5 ± 1.1 c	17.2 ± 0.4 c
	60 min	1778.9 ± 60.9 d	334.7 ± 16.6 d	97.8 ± 2.8 d	207.0 ± 2.6 d	2132.5 ± 18.9 d	71.8 ± 1.5 b	18.3 ± 0.1 b
	90 min	1619.0 ± 4.7 eB	268.8 ± 8.9 eB	59.0 ± 2.7 eB	199.0 ± 7.0 eB	2104.4 ± 12.9 dB	87.3 ± 1.1 aA	27.9 ± 3.0 aA
Corn oil (*C. longa* + corn oil)	0 min (control)	2577.5 ± 10.3 a	616.4 ± 5.5 a	241.4 ± 5.0 a	359.6 ± 10.5 a	2556.4 ± 59.8 a	44.9 ± 0.1 e	5.5 ± 0.3 e
	10 min	2527.2 ± 48.8 ab	578.6 ± 10.8 ab	206.9 ± 3.4 ab	292.1 ± 12.2 b	2493.9 ± 23.8 ab	45.9 ± 0.4 d	6.9 ± 0.1 d
	30 min	2219.0 ± 4.8 b	458.3 ± 1.7 b	134.9 ± 1.4 b	273.1 ± 2.8 c	2370.1 ± 18.6 b	61.0 ± 0.5 c	14.6 ± 0.4 c
	60 min	1958.4 ± 66.9 c	377.2 ± 12.1 c	99.2 ± 3.1 c	247.9 ± 3.6 d	2362.8 ± 52.3 c	68.3 ± 1.5 b	16.4 ± 0.2 b
	90 min	1818.7 ± 28.9 dA	336.8 ± 3.2 dA	83.0 ± 1.0 dA	209.7 ± 8.4 eA	2375.6 ± 47.2 cA	82.2 ± 0.7 aB	25.1 ± 0.1 aB

Notes:
1. Values represent mean ± SD (n = 3).
2. Different lowercase letters within a column indicate significant differences among heating times within each matrix ($p < 0.05$). Different uppercase letters in the 90 min row indicate significant differences in the extent of reduction among matrices ($p < 0.05$), where A represents the smallest reduction.
3. Abbreviations: BC, bisacurone; BDMC, bisdemethoxycurcumin; DMC, demethoxycurcumin; CUR, curcumin; AT, *ar*-turmerone; VNL, vanillin; DHG, dehydrozingerone.

Table S5. Quantitative profiles of 15 predominant sesquiterpenes and related volatile compounds in *Curcuma longa* (Ulgeum) extracts after thermal exposure at 180 °C for various durations.

Heating condition	Heating time	α-curcum.	(-)-zingibe.	U1	β-bisabol.	β-sesqui.	β-elemen.	U2	U3	*ar*-turmero.	α-turmero.	gemacro.	β-turmero.	bisabolo.	(E)-atlant.	curcume.
**Retention time (min)**		13.74	14.1	14.36	14.46	14.93	17.32	17.46	18.66	19.84	20.06	21.14	21.58	23.75	25.41	28.15
**Experimental retention indices**		1482	1496	1505	1508	1523	1599	1602	1631	1659	1664	1690	1700	1740	1771	1841
Dry (*C. longa* only)	0 min (control)	421.3 ± 9.5	429.4 ± 11.3	-	105.6 ± 4.4	545.5 ± 15.0	81.6 ± 8.9	28.4 ± 1.4	91.3 ± 1.6	2562.2 ± 104.0	2197.5 ± 80.6	575.3 ± 25.9	1226.9 ± 149.7	221.3 ± 15.3	529.4 ± 25.1	711.5 ± 35.0
	10 min	385.8 ± 4.5	384.0 ± 4.6	-	96.2 ± 0.3	496.5 ± 7.2	102.8 ± 6.5	80.2 ± 2.6	107.0 ± 9.9	2611.3 ± 42.2	2172.9 ± 27.6	583.4 ± 5.0	1171.9 ± 27.1	241.1 ± 2.9	538.9 ± 9.8	722.7 ± 22.7
	30 min	356.6 ± 5.8	341.7 ± 5.8	15.7 ± 0.8	89.5 ± 1.4	451.3 ± 6.1	199.4 ± 3.5	261.3 ± 6.0	96.0 ± 4.6	2416.0 ± 58.2	1815.8 ± 40.1	469.0 ± 17.2	1002.8 ± 23.4	226.1 ± 7.6	283.4 ± 8.8	522.5 ± 12.5
	60 min	337.1 ± 7.1	269.9 ± 6.0	31.5 ± 0.5	85.6 ± 1.6	367.7 ± 4.8	317.2 ± 1.3	247.4 ± 4.3	59.2 ± 3.1	2341.9 ± 26.3	1159.4 ± 14.5	245.6 ± 4.7	685.3 ± 13.8	174.0 ± 2.1	150.5 ± 3.2	402.6 ± 6.6
	90 min	334.5 ± 9.3	267.0 ± 9.8	31.6 ± 2.1	85.6 ± 3.9	358.4 ± 14.2	300.1 ± 20.6	238.5 ± 16.8	55.2 ± 5.6	2216.3 ± 109.9	1097.8 ± 57.4	231.3 ± 11.5	647.2 ± 38.6	170.9 ± 9.5	142.7 ± 8.8	369.2 ± 31.7
Water (*C. longa* + water)	0 min (control)	86.8 ± 3.9	81.5 ± 2.0	-	18.8 ± 1.5	101.2 ± 4.1	41.2 ± 0.2	15.4 ± 1.5	122.4 ± 1.6	1083.8 ± 67.3	746.9 ± 45.8	278.9 ± 13.0	403.3 ± 31.2	90.7 ± 7.7	393.7 ± 33.7	676.3 ± 62.8
	10 min	70.4 ± 3.3	62.9 ± 1.7	-	13.1 ± 1.5	76.7 ± 3.0	61.6 ± 9.9	18.8 ± 0.9	110.9 ± 9.8	1264.7 ± 71.8	800.1 ± 43.8	335.8 ± 14.2	435.4 ± 27.9	112.4 ± 8.5	489.0 ± 34.3	855.5 ± 56.8
	30 min	9.5 ± 0.4	6.3 ± 0.9	-	-	5.9 ± 0.3	6.3 ± 0.9	-	-	146.3 ± 4.5	84.4 ± 2.4	33.6 ± 1.2	53.0 ± 2.2	23.3 ± 2.2	220.4 ± 11.1	523.6 ± 36.3
	60 min	8.4 ± 1.0	7.4 ± 0.8	-	-	7.5 ± 0.6	-	-	-	10.1 ± 0.8	9.3 ± 1.3	-	5.2 ± 0.1	-	-	20.2 ± 1.8
	90 min	3.9 ± 0.4	10.2 ± 0.5	-	-	6.0 ± 0.2	-	-	-	6.0 ± 0.2	8.6 ± 0.5	-	2.6 ± 0.2	-	-	120.5 ± 6.0
Olive oil (*C. longa* + olive oil)	0 min (control)	537.9 ± 1.4	541.0 ± 0.4	-	129.3 ± 0.6	712.1 ± 8.2	105.3 ± 1.7	37.2 ± 3.2	115.7 ± 9.8	3366.3 ± 32.6	2898.6 ± 28.3	749.5 ± 11.6	1517.1 ± 28.7	267.0 ± 5.1	692.7 ± 2.9	861.1 ± 13.8
	10 min	481.4 ± 8.9	475.5 ± 8.5	-	110.2 ± 2.1	596.5 ± 13.6	183.1 ± 10.8	51.6 ± 0.3	117.9 ± 9.9	2976.8 ± 89.7	2538.7 ± 74.7	571.3 ± 8.6	1312.0 ± 37.1	238.6 ± 8.7	558.1 ± 19.5	803.5 ± 46.3
	30 min	537.6 ± 10.9	499.8 ± 7.3	3.5 ± 0.9	119.6 ± 3.0	639.8 ± 20.2	544.9 ± 32.3	130.7 ± 6.8	135.3 ± 9.7	3071.7 ± 185.0	2552.9 ± 153.5	251.5 ± 13.4	1319.2 ± 91.5	243.1 ± 16.7	369.7 ± 41.8	763.6 ± 84.2
	60 min	560.2 ± 12.9	355.8 ± 9.3	9.1 ± 1.2	112.8 ± 2.4	468.2 ± 7.1	697.5 ± 27.2	255.4 ± 9.4	132.2 ± 13.9	2916.1 ± 165.9	1556.5 ± 98.0	31.1 ± 1.8	906.0 ± 57.2	216.2 ± 14.5	193.6 ± 15.0	603.2 ± 46.6
	90 min	571.5 ± 6.7	287.0 ± 3.8	13.1 ± 1.1	111.2 ± 2.1	399.0 ± 0.6	671.0 ± 12.8	298.4 ± 10.9	120.7 ± 6.3	2888.5 ± 29.5	1208.3 ± 24.3	33.9 ± 2.1	751.5 ± 5.2	208.8 ± 7.2	164.3 ± 7.3	535.4 ± 18.6

Corn oil (*C. longa* + corn oil)	0 min (control)	530.1 ± 3.8	548.4 ± 1.9	-	119.4 ± 3.1	670.4 ± 0.5	42.0 ± 2.8	29.3 ± 1.0	19.8 ± 0.5	3294.4 ± 31.0	2914.2 ± 47.2	750.5 ± 7.4	1357.4 ± 7.8	233.4 ± 3.0	540.7 ± 9.1	711.5 ± 18.5
	10 min	523.6 ± 2.5	528.3 ± 11.1	-	114.0 ± 7.9	629.2 ± 4.4	115.2 ± 2.3	47.6 ± 3.3	53.3 ± 0.0	3239.0 ± 10.5	2788.3 ± 1.1	645.7 ± 30.3	1315.1 ± 76.3	228.6 ± 4.9	494.2 ± 40.1	728.9 ± 10.3
	30 min	530.2 ± 6.2	504.3 ± 8.7	4.1 ± 0.2	111.3 ± 2.6	592.2 ± 12.4	494.3 ± 0.5	127.6 ± 0.5	213.8 ± 1.8	3103.1 ± 78.4	2486.6 ± 60.9	240.8 ± 8.0	1221.7 ± 22.2	214.7 ± 38.6	305.8 ± 15.3	659.4 ± 4.3
	60 min	574.5 ± 14.1	367.6 ± 7.2	10.2 ± 0.6	98.2 ± 8.1	467.6 ± 0.7	676.8 ± 2.6	250.3 ± 6.6	313.8 ± 15.2	3009.0 ± 1.7	1568.4 ± 43.5	9.8 ± 0.8	844.5 ± 22.2	186.6 ± 7.3	146.3 ± 0.9	512.1 ± 20.6
	90 min	587.1 ± 8.9	294.1 ± 2.1	14.0 ± 0.3	100.2 ± 4.1	390.5 ± 2.3	657.7 ± 2.1	300.1 ± 7.9	308.9 ± 1.0	2983.6 ± 29.0	1226.6 ± 8.1	-	683.6 ± 61.4	174.6 ± 5.9	108.0 ± 2.5	459.7 ± 3.9

Notes:

1. Abbreviations: α-Curcum. (α-Curcumene), (−)-Zingibe. ((−)-Zingiberene), U1–U3 (Unknown; not structurally identified), β-Bisabol. (β-Bisabolene), β-Sesqui. (β-Sesquiphellandrene), β-Elemen. (β-Elemene), *ar*-Turmero. (*ar*-Turmerone), α-Turmero. (α-Turmerone), Gemacro. (Gemacrone), β-Turmero. (β-Turmerone), Bisabolo. (Bisabolone), (E)-Atlant. ((E)-Atlantone), Curcume. (Curcumenone).
2. Values are expressed as milligrams per kilogram ar-turmerone equivalents and represent mean ± SD (n = 3).
3. Experimental retention indices (RIs) were calculated using a homologous series of *n*-alkanes (C8–C24) analyzed on a DB-5MS column under identical chromatographic conditions. Compound identification was based on mass spectral matching against the NIST library and comparison of the experimental RIs with literature values.

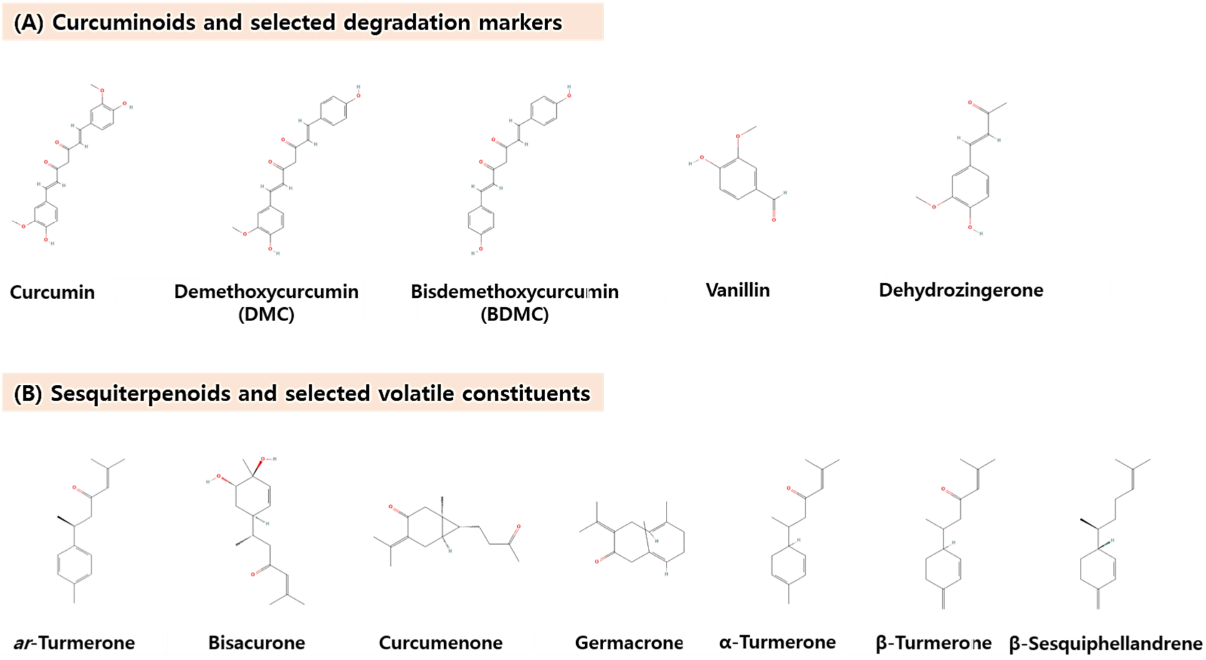

Figure S1. Chemical structures of the major phytochemicals and selected thermal degradation markers investigated in *Curcuma longa*. (A) Curcuminoids and selected degradation markers: curcumin, demethoxycurcumin (DMC), bisdemethoxycurcumin (BDMC), vanillin, and dehydrozingerone. (B) Sesquiterpenoids and selected volatile constituents: *ar*-turmerone, bisacurone, β-sesquiphellandrene, germacrone, curcumenone, β-turmerone, and α-turmerone. Chemical structures were obtained from the PubChem database (https://pubchem.ncbi.nlm.nih.gov/).
